# Design and fabrication of customized brain slice matrices using CAD and 3D printing technology

**DOI:** 10.1371/journal.pone.0317616

**Published:** 2025-01-17

**Authors:** Yosuke Yamazaki, Maki Yuguchi, Bin Honjo, Keitaro Isokawa

**Affiliations:** 1 Department of Anatomy, Nihon University School of Dentistry, Tokyo, Japan; 2 Division of Functional Morphology, Dental Research Center, Nihon University School of Dentistry, Tokyo, Japan; 3 Division of Oral Structural and Functional Biology, Nihon University Graduate School of Dentistry, Tokyo, Japan; IUBAT - International University of Business Agriculture and Technology, BANGLADESH

## Abstract

This study presents a novel method for creating customized brain slice matrices using Computer-Aided Design (CAD) and 3D printing technology. Brain Slice Matrices are essential jigs for the reproducible preparation of brain tissue sections in neuroscience research. Our approach leverages the advantages of 3D printing, including design flexibility, cost-effectiveness, and rapid prototyping, to produce custom-made brain matrices based on specific morphometric measurements. The developed protocol is user-friendly and incorporates features such as embossed identifying markers and support structures for challenging thin regions, thereby enhancing its practical utility. Our method demonstrates rapid and cost-effective fabrication of custom brain matrices, significantly reducing both material expenses and production time compared to traditional manufacturing techniques and ready-made products. This work contributes to the growing application of 3D printing in biomedical research, offering a valuable tool for neuroscientists requiring precise and consistent brain tissue sectioning.

## Introduction

Additive Manufacturing (AM), commonly known as 3D printing, has emerged as a transformative technology in the era of Industry 4.0, revolutionizing various sectors, including healthcare, aerospace, and manufacturing [1, 2]. This innovative process allows for the creation of complex three-dimensional objects by depositing materials layer by layer, offering advantages such as reduced material waste, increased design flexibility, and the ability to produce customized parts.

3D printing technology has found diverse applications in scientific research, revolutionizing various fields. In biomedical engineering, 3D bioprinting enables the creation of complex tissue constructs and organ models, advancing regenerative medicine and drug development [3, 4]. Additionally, 3D printing facilitates the rapid prototyping of customized laboratory equipment and the fabrication of intricate experimental setups [5]. These applications demonstrate how 3D printing is accelerating scientific progress by offering unprecedented flexibility, precision, and speed in creating research tools and models across multiple disciplines.

In this study, we developed a method to create a jig for brain sectioning, known as a Brain Slice Matrix, using Computer-Aided Design (CAD) and 3D printing technology for the reproducible preparation of brain tissue sections. This approach enables the consistent production of thin brain slices for experimental purposes. Brain matrices, serving as jigs for tissue sectioning, are commercially available, and applications of 3D printing technology for their fabrication have already been documented [6]. In our approach, brain matrix shapes based on morphometric measurements are generated using CAD software, and these shapes are then 3D printed to manufacture custom-made brain matrices.

## Materials and methods

### Animal experiments

An adult C57BL/6 mouse was anesthetized via intraperitoneal injection with a combination of three anesthetic agents: medetomidine hydrochloride (0.3 mg/kg), midazolam (4 mg/kg), and butorphanol tartrate (5 mg/kg). The depth of anesthesia was confirmed by observing the absence of the pedal withdrawal reflex, along with a reduction in respiratory rate due to abdominal breathing and the loss of the corneal reflex. Once deep anesthesia was achieved, the mouse was decapitated, and the brain was extracted. The dimensions of the extracted brain were then measured.

### Brain matrix fabrication

The protocol described in this peer-reviewed article is published on protocols.io, https://dx.doi.org/10.17504/protocols.io.261ge563jg47/v1 and is included for printing as S1 File with this article. The code was executed in OpenSCAD (version 2021.01; https://openscad.org) to generate the Brain Matrix data. This study employed a Form 2 3D printer (Formlabs, Boston, USA), PreForm software (version 3.38; Formlabs), and Gray Resin v4 (Formlabs) as the printing material. After fabrication, the brain matrix was rinsed with isopropyl alcohol (IPA) and then cleaned in an ultrasonic cleaner while immersed in IPA, with the IPA replaced three times during the process. Subsequently, the remaining liquid between the slots was thoroughly removed using a blower. After air drying, secondary curing was performed under UV light. The secondary curing was carried out using a Form Cure (FormLabs) at 60°C for 30 minutes. Photographs of the fabricated Brain Matrix are provided in Supporting Information file (S1 File).

### Ethical statement

The experimental procedures were approved by the Institutional Animal Care and Use Committee of Nihon University School of Dentistry (AP18DEN028-2). All procedures were conducted according to the Fundamental Guidelines for Proper Conduct of Animal Experiments and Related Activities in Academic Research Institutions issued by the Japanese Ministry of Education, Culture, Sports, Science, and Technology.

## Results and discussion

**Fig 1:**
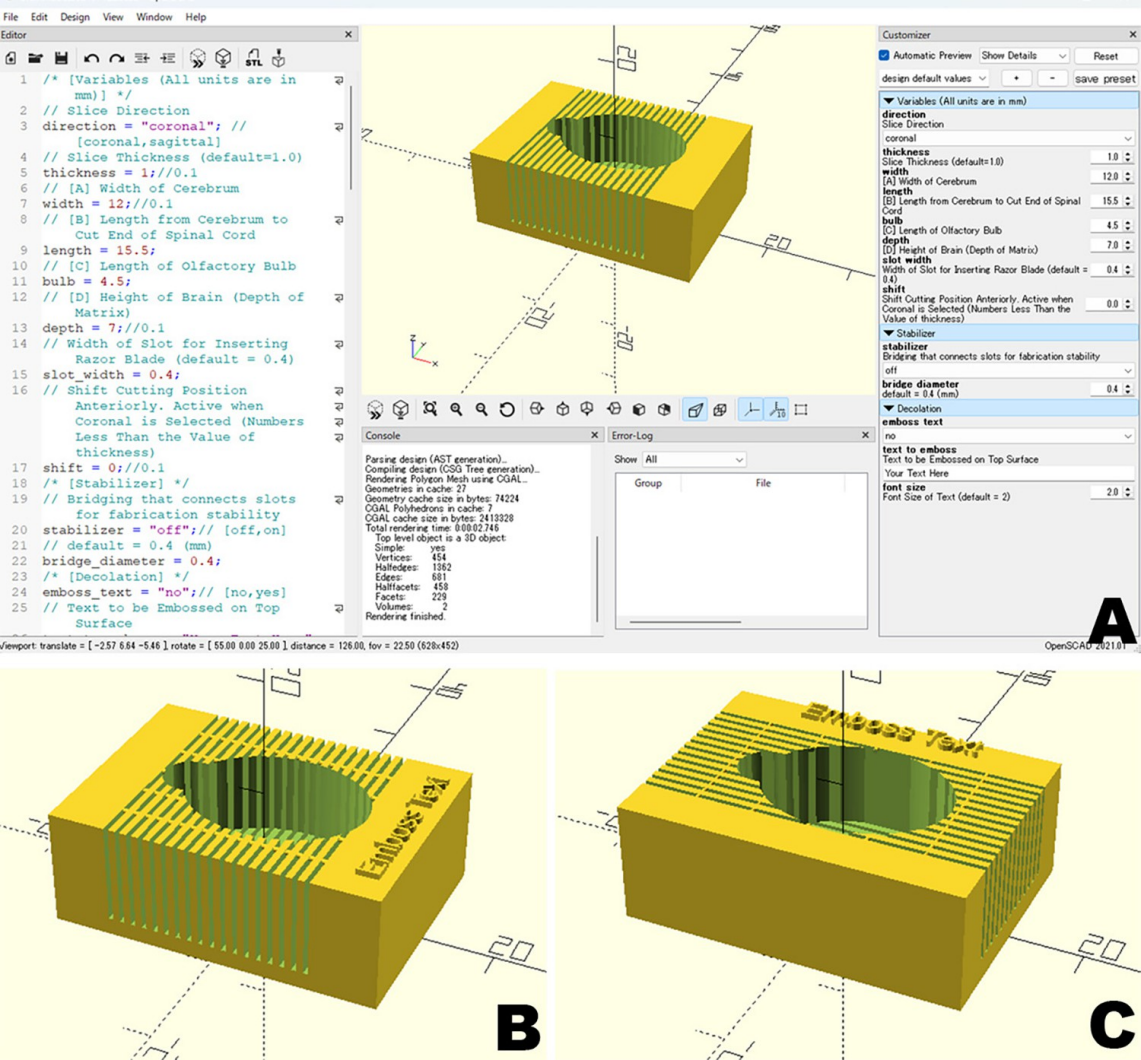
Interface for brain matrix creation in OpenSCAD. (A) The Brain Matrix generated according to the loaded code is displayed in the center of the screen. By adjusting the parameters on the right side of the screen, the design of the Brain Matrix is updated accordingly. In this way, users can create Brain Matrix data tailored to variations in brain size, slice thickness, and other parameters in a parametric manner without modifying the code. Examples of a Brain Matrix with sample names embossed: a coronal section (B) and a sagittal section (C).

The brain matrix described in the protocol was successfully fabricated using 3D printing technology. The CAD program code included features for embossing identifying strings or numbers on the constructed model, as well as mechanisms to support thin structures that were challenging to print, making it immediately practical for use (Fig 1). This method demonstrated significant advantages in terms of both cost-effectiveness and fabrication speed (Table 1).

**Table 1:** Results and specifications of 3D-printed mouse brain matrix fabrication.

Parameter	Details
Tissue sample	Adult C57BL/6 mouse brain
Tissue weight	0.54 g
Fabricated brain matrix dimensions	20 × 27 × 10 mm
Resin volume used	6.01 mL (including supporting structures)
Estimated cost	Approximately 156 JPY (using Gray Resin v4), calculated based on a 1L cartridge price of 26,000 JPY
Fabrication time	2 hours 9 minutes at 0.05 mm layer height

The fabrication time represents only the duration of the 3D printing process and does not include post-processing steps such as washing or post-curing. JPY, Japanese Yen

A brain matrix designed for sectioning the brain of an adult C57BL/6 mouse required approximately 6 ml of resin for fabrication. This resulted in material costs of approximately 1 US dollar per unit. Using Form 2 and Gray Resin, the fabrication time was approximately 2 hours with a layer thickness setting of 0.05 mm. These parameters demonstrate exceptional cost-effectiveness and rapid production capabilities, making this approach highly advantageous for researchers requiring custom brain matrices. In this study, we used resin commonly employed for model fabrication. While this choice was considered sufficient in terms of precision, selecting a different resin might be necessary from the perspective of reusability. Fabrication using Gray Resin (Formlabs) allows for cleaning with alcohol; however, single-use may be preferable. High-strength resins and resins compatible with autoclaving are commercially available, suggesting the possibility of applying such materials for future fabrications. AM is considered the most cost-effective approach for producing custom-made parts, particularly in a laboratory setting for fabricating brain matrices. This is especially true for applications requiring low-volume, high-variety production, where the flexibility of AM aligns well with the specific needs of experimental research. It is particularly effective for fabricating brain matrices tailored to brain tissues of sizes or types not covered by commercially available products, or for research focusing on developmental stages where the size of the brain changes over time.

While this study focused on mouse brain applications, minor modifications and revisions to the CAD program could extend its utility to other animal species or different organs, highlighting the versatility of this method. By incorporating modifications that account not only for differences in brain size but also for variations in the morphology of specific regions, this method could potentially be applied to the brains of other animal species. Such modifications could be implemented through adjustments to the CAD code, allowing for precise customization to accommodate different anatomical features. Additionally, volumetric data obtained from CT or MRI scans could be utilized to subtract the brain volume, as well as the structure of other organs or even whole-mount specimens in the case of small animals, from a cubic template. This would enable the design of a negative mold tailored to these diverse structures, highlighting the flexibility and broad applicability of the method.

In conclusion, AM has enabled the in-laboratory fabrication of custom-made devices without the need for large, complex machinery typically required for commercially available products. This approach leverages key advantages such as low cost and high flexibility in design and manufacturing, allowing researchers to tailor devices to their specific experimental needs [7, 8]. While the precision and reproducibility of the fabricated products depend on the performance of the AM equipment and the materials used, AM is particularly well-suited for creating tools that assist in experiments, such as jigs or hand instrumentation aids. These applications present minimal barriers for implementation, making AM a practical and accessible solution for experimental research.

## Supporting information

S1 File Step-by-step protocol, also available on protocols.io. (PDF)
