# Supplementary material for: Design and fabrication of customized brain slice matrices using CAD and 3D printing technology
Source: PLoS One. 2025 Jan 17;20(1):e0317616. doi: 10.1371/journal.pone.0317616 (PMC11741617; doi:10.1371/journal.pone.0317616)
Supplement: S1 File — (PDF) [file pone.0317616.s001.pdf]

Aug 06, 2024

# Creating Custom Brain Slicing Matrices with 3D Printers

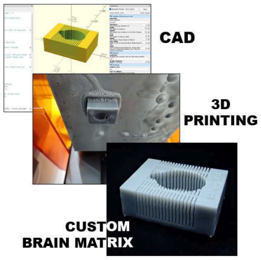


DOI
dx.doi.org/10.17504/protocols.io.261ge563jg47/v1

Yosuke Yamazaki[1,2,3], Maki Yuguchi[1,2,3], Keitaro Isokawa[1,2,3]

[1]Department of Anatomy, Nihon University School of Dentistry;
[2]Division of Functional Morphology, Dental Research Center, Nihon University School of Dentistry;
[3]Division of Oral Structural and Functional Biology, Nihon University Graduate School of Dentistry

Yosuke Yamazaki
Nihon University School of Dentistry

OPEN ACCESS

**DOI:** dx.doi.org/10.17504/protocols.io.261ge563jg47/v1

**Protocol Citation:** Yosuke Yamazaki, Maki Yuguchi, Keitaro Isokawa 2024. Creating Custom Brain Slicing Matrices with 3D Printers. **protocols.io** https://dx.doi.org/10.17504/protocols.io.261ge563jg47/v1

**License:** This is an open access protocol distributed under the terms of the **Creative Commons Attribution License**, which permits unrestricted use, distribution, and reproduction in any medium, provided the original author and source are credited

**Protocol status:** Working
**We use this protocol and it's working**

**Created:** March 28, 2024

**Last Modified:** August 13, 2024

**Protocol Integer ID:** 97478

**Keywords:** Tissue Sectioning, Brain Matrix, Brain Slicer

**Funders Acknowledgements:**
**Grant from Dental Research Center, Nihon University School of Dentistry**
Grant ID: DRC(B)-2024-2, DRC(B)-2023-2, 2022
**Sato Fund, Nihon University School of Dentistry**
Grant ID: SATO-2024-2, SATO-2023-2, 2022

## Disclaimer

The **protocols.io** team notes that research involving animals must be conducted according to internationally-accepted standards and should always have prior approval from an Institutional Ethics Committee or Board.

## Abstract

To create reproducible brain sections, brain slicing matrices are often utilized. These matrices are designed with anatomically proportioned cavity dimensions and precisely spaced slice channels, enabling consistent and accurate slicing of the brain into coronal or sagittal sections. Brain matrices can be customized for various animal species or developmental stages, and a wide range of models are commercially available. However, there are certain limitations to the variety of these prefabricated products. In this protocol, we design a brain matrix specifically for rodents, tailored to the measurements of the brain sample, and produce the customized brain slicing matrix with a 3D printer.

## Materials

Software
- OpenSCAD version 2021.01
- Slicer software (ex. PreForm for Form2)

Equipments
- 3D printer (For accuracy, an SLA 3D printer is preferred)
- UV curing machine
- Forceps
- Diagonal cutting pliers
- Beakers
- Disposable gloves, protective masks for organic solvents, and safety goggles

Materials
- UV-curring resin material
- Washing solution (Organic solvents such as isopropyl alcohol)

## Mesuratent of the sample

1 The mouse brain sample is placed on the dissection mat.

2 Following the specified metrics, the approximate length is measured.

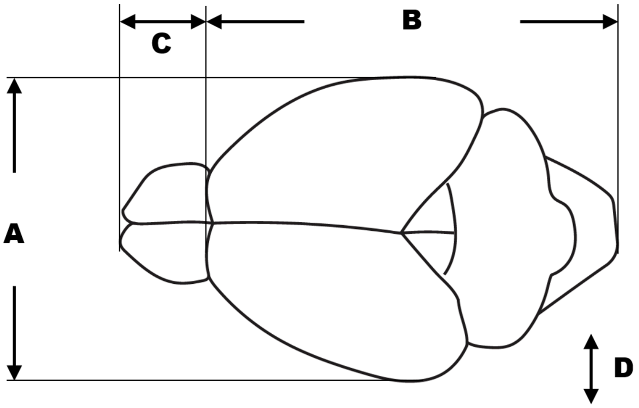


Schematic diagram of the dorsal view of the mouse brain. The measurement points are as follows: (A) Width of the cerebrum, (B) Length from the cerebrum to the cut end of the spinal cord, (C) Length of the olfactory bulb, and (D) Height of the brain (depth of the matrix).

## Installation of OpenSCAD

3 For Windows, download the installer from **https://openscad.org/** and execute the .exe file to proceed with the installation, or use the following command in the terminal:

```
winget install --id OpenSCAD.OpenSCAD
```

4 For macOS, download the dmg file from **https://openscad.org/** and install OpenSCAD program from it, or, on a system with Homebrew enabled, use the following command in the terminal:

```
brew install openscad
```

## Excute the code for generation

5 Run the OpenSCAD program.

6 Choose New button for a new file creation.

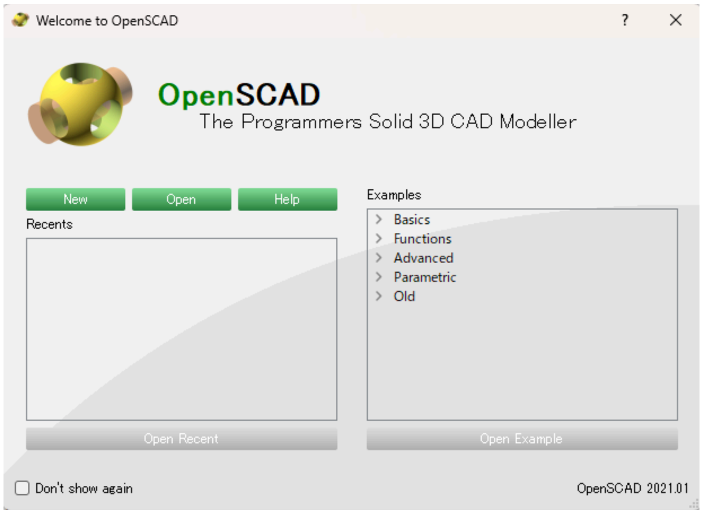


Initial screen of OpenSCAD. Start by pressing the "New" button on the left side.

7 Copy and paste the code below to the Editor pane on the left side of OpenSCAD window.

```
/* [Variables (All units are in mm)] */
// Slice Direction
direction = "coronal"; // [coronal,sagittal]
// Slice Thickness (default=1.0)
thickness = 1;//0.1
// [A] Width of Cerebrum
width = 12;//0.1
// [B] Length from Cerebrum to Cut End of Spinal Cord
length = 15.5;
// [C] Length of Olfactory Bulb
bulb = 4.5;
// [D] Height of Brain (Depth of Matrix)
depth = 7;//0.1
// Width of Slot for Inserting Razor Blade (default = 0.4)
slot_width = 0.4;
// Shift Cutting Position Anteriorly. Active when Coronal is
Selected (Numbers Less Than the Value of thickness)
shift = 0;//0.1
/* [Stabilizer] */
// Bridging that connects slots for fabrication stability
stabilizer = "off";// [off,on]
// default = 0.2 (mm)
bridge_diameter = 0.2;
/* [Decolation] */
emboss_text = "no";// [no,yes]
// Text to be Embossed on Top Surface
text_to_emboss = "Your Text Here";
// Font Size of Text (default = 2)
font_size = 2;//0.1
// Calculating dimensions for the outer box
outer_length = length + bulb + 8;
outer_width = width + 8;
outer_height = depth + 3;

module makeOuterbox(){
    cube([outer_length, outer_width, outer_height], center=true);
}

module drawBrainOutline() {
        translate([bulb/4, 0, outer_height/2 - depth/2]) {
            scale([length/width, 1, 1]) cylinder(h=depth,
r=width/2, $fn=100, center=true);
            translate([-length/2 + bulb/2, 0, 0]) cube([bulb,
bulb, depth], center=true);
```

```
            translate([-length/2, 0, 0]) cylinder(h=depth,
r=bulb/2, $fn=100, center=true);
    }
}

module makeSlot() {
    if (direction == "coronal") {
        translate([bulb/4, 0, outer_height/2 - depth/2]) {
            for (x = [-(length+bulb)/2 - shift: thickness :
length/2]) {
                translate([x, 0, 0])
                    cube([slot_width, outer_width, depth + 3],
center=true);
            }
        }
    }
    else if (direction == "sagittal") {
        translate([0, 0, outer_height - depth - 1])
cube([outer_length, slot_width, depth + 3], center=true); //
Center groove
        for (i = [0 : thickness : floor(width/2)]) {
            translate([0, i, outer_height - depth - 1])
cube([outer_length, slot_width, depth + 3], center=true);
            translate([0, -i, outer_height - depth - 1])
cube([outer_length, slot_width, depth + 3], center=true);
        }
    }
}

module embossText() {
    if (emboss_text == "yes") {
        if (direction == "coronal") {
            translate([outer_length/2 - bulb/4, 0, outer_height])
{
                rotate([0, 0, 90])
                linear_extrude(height=.5)
                    text(text_to_emboss, size=font_size,
halign="center", valign="bottom");
            }
        } else if (direction == "sagittal") {
            translate([0, outer_width/2, outer_height]) {
                rotate([0, 0, 0])
                linear_extrude(height=.5)
                    text(text_to_emboss, size=font_size,
halign="center", valign="top");
```

```
            }
        }
    }
}

module addStabilizer() {
    if (stabilizer == "on") {
        if (direction == "coronal") {
            for (y = [(-outer_width/2)*2/3, (-outer_width/2)/3, 0,
(outer_width/2)/3, (outer_width/2)*2/3]) {
                translate([0, y, outer_height/2 -
bridge_diameter/2])
                    rotate([0, 90, 0])
                        cylinder(h=outer_length,
d=bridge_diameter, center=true, $fn=20);
            }
        } else if (direction == "sagittal") {
            for (x = [(-outer_length/2)*2/3, (-outer_length/2)/3,
0, (outer_length/2)/3, (outer_length/2)*2/3]) {
                translate([x, 0, outer_height/2 -
bridge_diameter/2])
                    rotate([90, 0, 0])
                        cylinder(h=outer_width, d=bridge_diameter,
center=true, $fn=20);
            }
        }
    }
}

translate([0, 0, outer_height/2]) {
    difference() {
        union() {
            difference() {
                makeOuterbox();
                makeSlot();
            }
            addStabilizer();
        }
        drawBrainOutline();
    }
}
embossText();
```

Review the 3D preview and, if necessary, return to the previous steps to adjust the parameters.

8 Change the value of the parameters shown below based on the measurement results. The input field is located in the Customizer pane on the right side of the window.

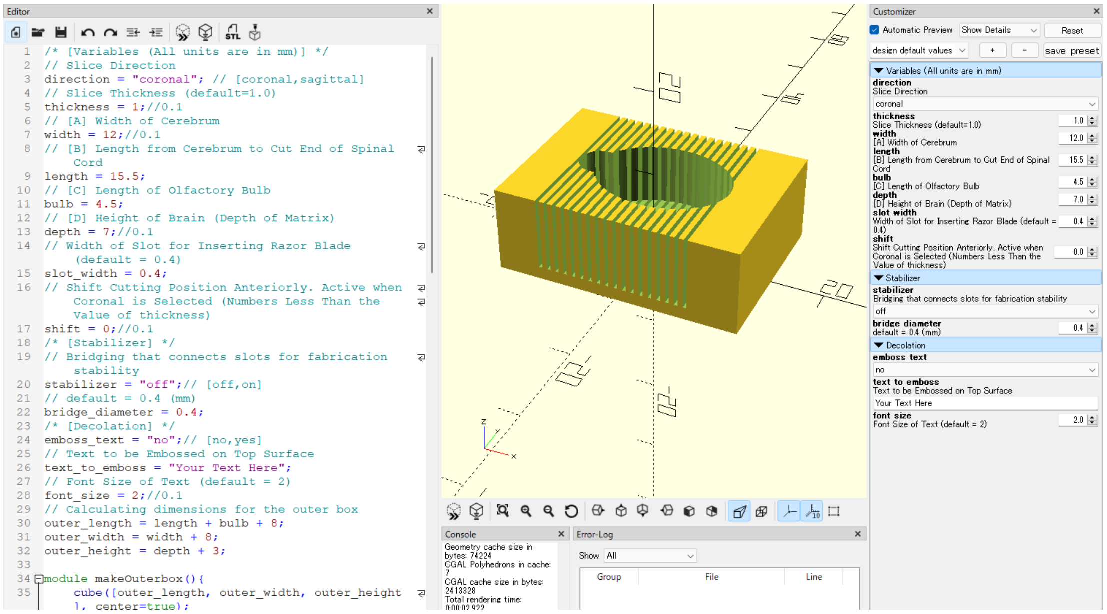


OpenSCAD interface.

The parameters are:

8.1 **direction**: Choose either "sagittal" or "coronal". The former should be selected when creating slices that section the sample sagittally, while the latter is chosen for slices that section the sample coronally.

8.2 **thickness**: The value indicates the slice thickness. The default value is set at 1 (mm). To specify a slice thickness of 2mm, enter 2.

8.3 **witdth, length, bulb and depth**: Input values that are 10% larger than the measurements obtained in the previous step.

8.4 **slot width**: This item specifies the width of the slot for inserting a razor blade. Although the actual thickness of the razor is thinner, reducing the width of the slot excessively can make the fabrication process challenging.

8.5 **shift**: When "coronal" is selected and adjustment of the slice position is desired, the slots will shift anteriorly by the specified value of the shift setting. Enter a value that is less tha the value set for thickness.

8.6 **stabilizer**: Add thin rod-like structures that bridge the slots. These contribute to the fabrication stability of narrow slots.

8.7 **bridge diameter**: Define the diameter of the bridge structure as a stabilizer. If the default value of 0.4mm or a similar value is used, it can be easily cut with a razor blade.

8.8 **emboss text**: Choose either "no" or "yes". Upon selecting "yes," the designated text will be displayed at the top of the matrix.

8.9 **text to emboss**: Enter the text or numerical string. A long text will not fit within the designated space.

8.10 **font size**: The font size for the embossing text can be set.

9 Push the Preview button on the top of the Editor pane. The outline of the matrix is updated based on the specified values. Review the 3D preview and, if necessary, return to the previous steps to adjust the parameters.

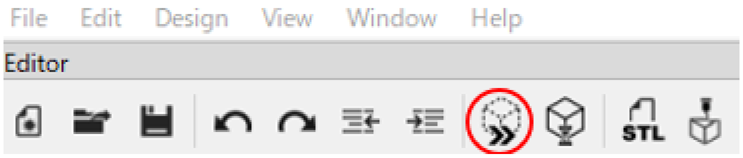


Icons located at the top of the Editor pane. The button circled in red is the Preview button.

10 Pressing the Render button displays the designed brain matrix on the screen. It may take several seconds for the rendering results to be displayed.

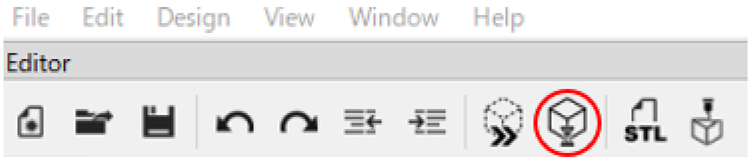


Icons located at the top of the Editor pane. The button circled in red is the Render button.

## Obtain the STL file

11 Push the Export to STL icon on the top of the Editor pane.

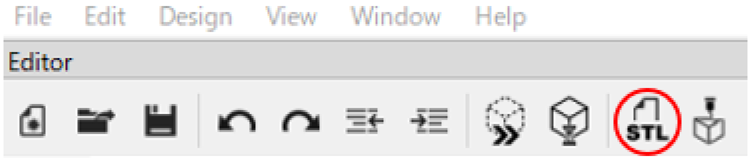


Icons located at the top of the Editor pane. The button circled in red is the Export to STL button.

A dialog box will appear asking for the save location of the STL file. Select the desired save location and click OK.

## Digital Fabrication

12 Load the STL file into slicer software compatible with the 3D printer. In the following steps, protocols will be provided for using the PreForm to fabricate the design with Formlabs' Form2 printer.

13 Run PreForm.

14 In the menu bar, click **File** > **Open**. The Open File window appears. Select the STL file and click OK.

15 Configure the orientation of the 3D model and the settings for support structures.

16 Select the printer model, material, and layer thickness.

**Note**

The smallest slice thickness does not necessarily result in the best fabrication. The optimal slice thickness should be determined by considering the balance between fabrication speed and accuracy.

## Post-processing after fabrication

17 Remove the object from the build plate and either peel off the support material by hand or cut it off with diagonal cutting pliers.

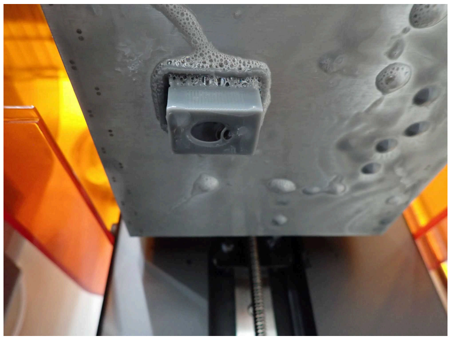

The condition immediately after printing is shown. The printed object attached to the build plate has uncured resin adhered to it.

**Safety information**

Uncured resin, which can be harmful, may adhere to the surface of the object. It is advisable to wear gloves to avoid direct contact and to use a mask to block the vapors of organic solvents.

18 Wash the object with isopropyl alcohol (IPA) for slightly longer than the recommended time for the resin used, in order to remove uncured resin from narrow slots. For more effective cleaning, the use of a magnetic stirrer or an ultrasonic cleaner is recommended.

19 Quickly dry the object after removing it from the cleaning solvents, and then proceed with post-curing. Use an air blower to remove any droplets from the narrow gaps.

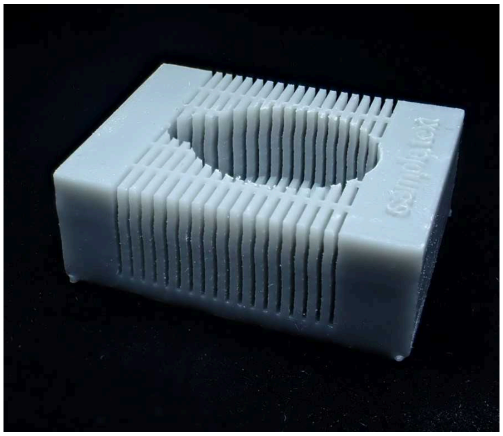

The brain slice matrix after waching and post-curing.
